# Potential of large language models for rapid clinical information support: evidence from acute kidney injury knowledge testing

**DOI:** 10.1038/s41598-026-46846-7

**Published:** 2026-04-02

**Authors:** Philipp Russ, Simon Bedenbender, Jonas Einloft, Hendrik L. Meyer, Leo T. Wenzel, Andre Ganser, Martin C. Hirsch, Ivica Grgic

**Affiliations:** 1 Institute for Artificial Intelligence in Medicine, Marburg University, University Hospital Giessen and Marburg, Marburg, Germany; 2 Department of Internal Medicine and Nephrology, Marburg University, University Hospital Giessen and Marburg, Marburg, Germany

**Keywords:** Artificial intelligence (AI), Acute kidney injury (AKI), Clinical decision support, Digital health, Large language model (LLM), Health care, Medical research, Nephrology

## Abstract

Large language models (LLMs) are increasingly applied in clinical contexts; however, to our knowledge, their performance in acute kidney injury (AKI) has not been directly compared with physician performance on standardized clinical knowledge assessments. We conducted a cross-sectional study to evaluate the performance of 13 publicly available LLMs and 123 volunteer participants at the 131st Annual Congress of the German Society of Internal Medicine in Wiesbaden, Germany. Both groups completed an identical AKI knowledge assessment consisting of two case vignettes and 15 single-best-answer multiple-choice questions. LLMs achieved a mean score of 13.5 out of 15 (90%), with several models reaching a perfect score, while human participants averaged 7.3 out of 15 (48.7%). Only 16.3% of participants scored 11 points or higher. As an illustrative example, ChatGPT-4o completed the test in approximately 0.5 minutes, whereas humans required a mean of 7.3 minutes. These findings demonstrate that LLMs substantially outperformed a heterogeneous group of medical professionals in AKI knowledge assessments and did so with markedly greater efficiency. While this highlights the potential of LLMs as rapid, cost-effective tools for clinical knowledge support, their role in real-world patient care remains undetermined, and human clinical judgment remains essential to ensure safe, context-sensitive, and patient-centered care.

**Supplementary Information:**

The online version contains supplementary material available at 10.1038/s41598-026-46846-7.

## Introduction

Acute kidney injury (AKI) is a common and clinically relevant complication in inpatient and emergency care settings. Depending on the patient cohort, it occurs in approximately 10–15% of individuals admitted to hospital and in up to 50% of those requiring intensive care^1^. Timely recognition and swift, accurate intervention are essential to avert irreversible damage and prevent the progression to serious complications including chronic kidney disease (CKD)^2,3^. Approximately 11–15% of the global population is affected by CKD^4^ and it is associated with increased mortality and morbidity^5^. Not only is the quality of life of affected patients significantly impaired^6^, but CKD also contributes to a substantial global economic burden^7^.

To support rapid clinical decision-making and reduce the risk of short- and long-term consequences, medical knowledge must be readily accessible at the point of care. In this context, artificial intelligence (AI), particularly large language models (LLMs), has gained increasing importance in medicine^8,9^. Previous studies have demonstrated the value of LLMs in diverse applications, such as clinical documentation, automated report generation, and patient communication^10–14^. More recently, research has begun exploring their role in supporting clinical decision-making in internal medicine, including oncology and gastroenterology^15–17^.

In nephrology, early investigations have explored the use of LLMs for knowledge support and clinical question answering^18,19^. However, systematic head-to-head comparisons between different LLMs and medical professionals in practice-oriented scenarios are still lacking. To address this gap, we developed and deployed an interactive test conducted at the 2025 annual congress of the German Society of Internal Medicine. This study evaluated the capacity of LLMs to accurately discern and apply factual knowledge in comparison with healthcare professionals, focusing specifically on AKI.

## Methods

### Selection of the LLMs

A total of 13 LLMs were included in the evaluation, representing a broad cross-section of currently available systems. Selection criteria aimed to capture diversity in model architecture, training approach, deployment modality and accessibility, based on publicly available information. The sample included both proprietary and open-access systems, instruction-tuned variants, and models developed by major commercial providers as well as open-source research organizations. Specifically, we evaluated the following LLMs (using the versions made available by their developers in April 2025): ChatGPT 4o (as of April 11, 2025)^20^, ChatGPT 4o-mini^21^, ChatGPT 4.5^22^, ChatGPT 4^23^, ChatGPT o3-mini-high and ChatGPT o3-mini (reasoning)^24^, Claude 3.7 Sonnet^25^, Gemini 2.0 Flash^26^, Gemini 2.5 Pro Experimental^27^, Mistral Small 3.1^28^, DeepSeek V3-0324^29^, DeepSeek R1^30^, and Grok-3^31^(Supplementary Table 1). All models were publicly available at the time of data collection. Evaluation was performed via each model’s official user interface or application programming interface (API), depending on availability and deployment context. To initiate the knowledge test, all models received the following prompt: “Please answer the questions and provide an overview of your answer options”. All models were tested using default settings. Both case vignettes and all 15 multiple-choice questions were submitted in a single prompt, and each model was evaluated once. As an exploratory follow-up, available models were additionally assessed with three prompt variations, designed to reflect neutral, role-based, and guideline-oriented instructions.

### Study design and participants

The concept of an “AI vs. Human” challenge was designed to compare the capacity of LLMs and humans in discerning factual knowledge on the topic of AKI. The activity was framed as a competition, with participants informed that 13 LLMs had just before completed the same test. The questionnaire included two brief nephrology case vignettes, each followed by a series of single-answer multiple-choice questions (see the Section Questionnaire Design for details).

**Fig. 1:**
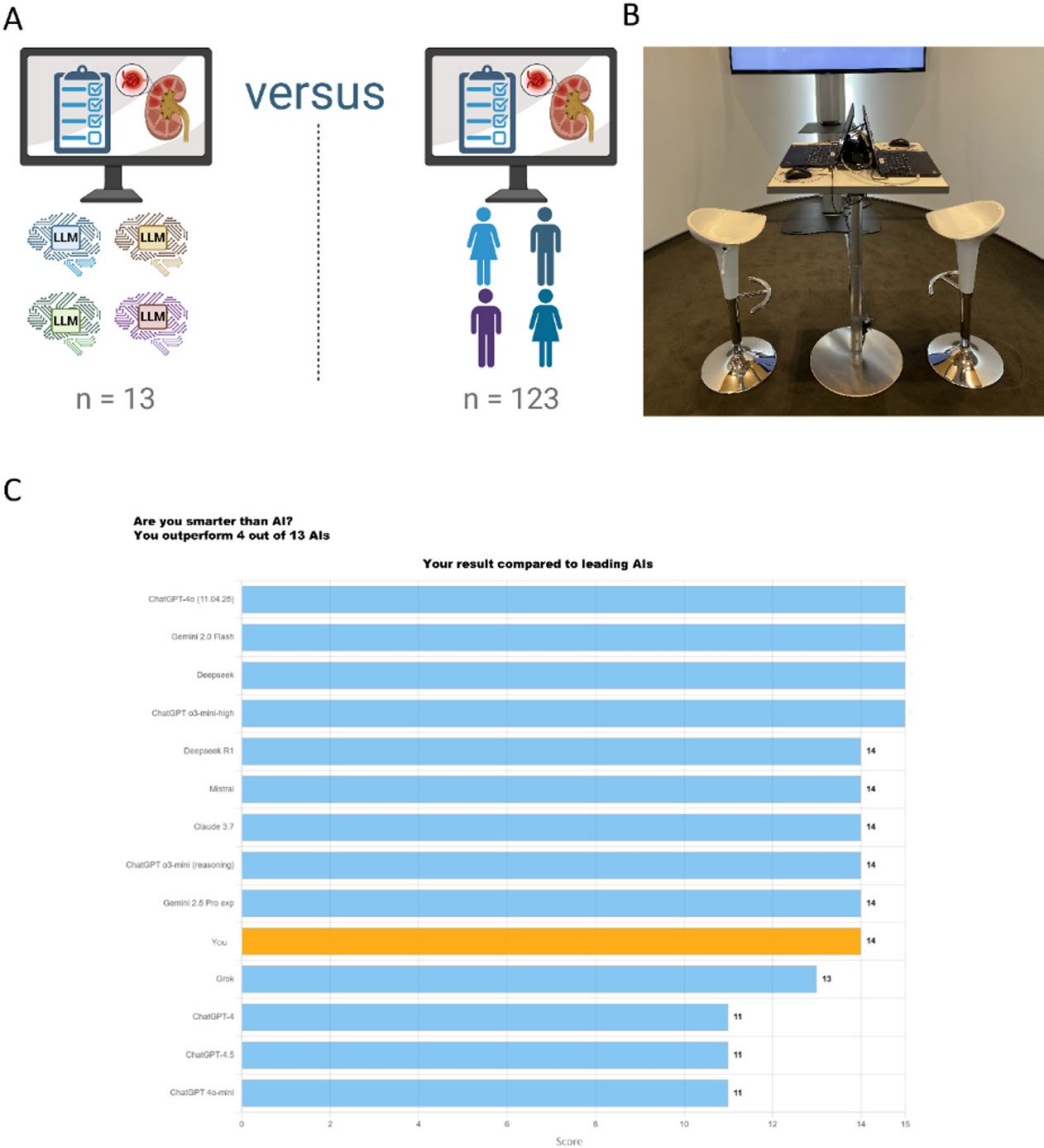
**(A)** Schematic illustration of the study concept. Volunteer congress attendees competed against 13 large language models (LLMs) in an acute kidney injury (AKI) knowledge test. **(B)** Study setting at the 131st Annual Congress of the German Society of Internal Medicine (Wiesbaden, Germany, May 2025). Two laptops at an open, self-service station providing access to the online test. **(C)** Example of feedback (rank) displayed to a participant upon test completion. The bar chart shows the participant’s score (orange) in comparison to the performance of all “participating” LLMs (blue). In this example, the participant achieved 14 out of 15 points, outperforming 4 of the 13 LLMs.

The study was conducted during the 131st Annual Congress of the German Society of Internal Medicine, held in Wiesbaden, Germany, from May 3 to 6, 2025, with approximately 9,000 attendees. The goal was to obtain a cross-sectional sample of internist physicians as well as medical students interested in a specialization in internal medicine. Participation was facilitated via an unstaffed, self-service station located in a semi-separated, designated part of the exhibition area. A large screen displayed an invitation to participate reading ‘Are you smarter than AI?’, while two laptops were positioned in front of the screen, providing access to the questionnaire hosted on the SoSci Survey platform (SoSci Survey GmbH, Munich, Germany). The “challenge” format was chosen to maximize visibility and encourage engagement among congress attendees. Technical assistance was available on demand and repeat participation by the same individual excluded. The study was conducted as a single cross-sectional observational investigation with exploratory intent and did not include longitudinal follow-up or external validation. The study setting is illustrated in Fig. 1A (created using BioRender) and Fig. 1B (photograph of actual setting).

### Consent to participate and ethics approval

Participants were required to provide informed consent by actively ticking a checkbox before accessing the questionnaire; the survey could not be initiated without consent. All responses were collected and analyzed in anonymized form. Ethical approval for the study was obtained from the Ethics Committee of the Faculty of Medicine, Marburg University (File Number “25–76 ANZ”). All procedures were conducted in accordance with the Declaration of Helsinki and in compliance with local regulations for human subject research.

### Questionnaire design

The questionnaire consisted of two clinical case vignettes and 15 single-answer multiple-choice questions, as well as items on demographics and professional background (gender, stage of medical training and career, and field of specialization). Both clinical cases and all questions pertained to AKI and its clinical implications. The questions adhered closely to the evidence and recommendations outlined in the KDIGO Clinical Practice Guideline for Acute Kidney Injury^32^, supplemented by nephrology knowledge routinely taught in medical training. The content was developed by two board-certified internists, including one board-certified nephrologist, both with experience in undergraduate and postgraduate medical education. To ensure clinical relevance and comprehensibility, the draft questionnaire underwent iterative internal expert review within the study team. During this process, the items were checked for guideline concordance, clarity of wording, appropriateness of difficulty, and the presence of a single best answer, and were refined until consensus was reached. The test began with a brief clinical vignette, followed by five multiple-choice questions, some directly related to the case and others independent. A second vignette was then presented, followed by ten additional questions in the same format. In total, participants could achieve a maximum score of 15 points. The questionnaire was administered in German to both human participants and the LLMs.

After completing the test, participants were asked to estimate the average performance of the LLMs. Finally, they were shown a comparison of their own score along with the LLMs results (Fig. 1C). The full text of the cases and questions is provided in Supplementary File S2.

### Data analysis and graphical illustration

Survey responses were collected using the SoSci Survey platform (SoSci Survey GmbH, Munich, Germany) and exported to Microsoft Excel (version 16, Redmond, WA, USA) for subsequent data analysis. Statistical analyses and graphical representations were performed using GraphPad Prism (version 10; Boston, MA, United States). The schematic illustration of the study design (Fig. 1A) was created using BioRender (Toronto, Canada).

Descriptive statistics included means, standard deviations (SD), medians, and interquartile ranges (IQR) to characterize central tendency and data dispersion. Data normality was assessed using the Shapiro–Wilk test. As the data were not normally distributed, group comparisons were conducted using the non-parametric Kruskal–Wallis test. Data are reported as mean ± SD or median with IQR, as appropriate.

## Results

### Baseline characteristics of study participants

A total of 123 congress attendees completed the challenge, comprising 56 women (45.5%), 59 men (48.0%), and 8 individuals (6.5%) who did not specify their gender. The median questionnaire completion time was 7.1 min (IQR: 5.4–9.0), with a mean of 7.3 ± 2.5 min.

By professional stage, 44 participants (35.8%) were medical students, 29 (23.6%) were resident physicians, 31 (25.2%) were board-certified specialists, 12 (9.8%) were attending physicians, and 7 (5.7%) were chief physicians.

**Fig. 2:**
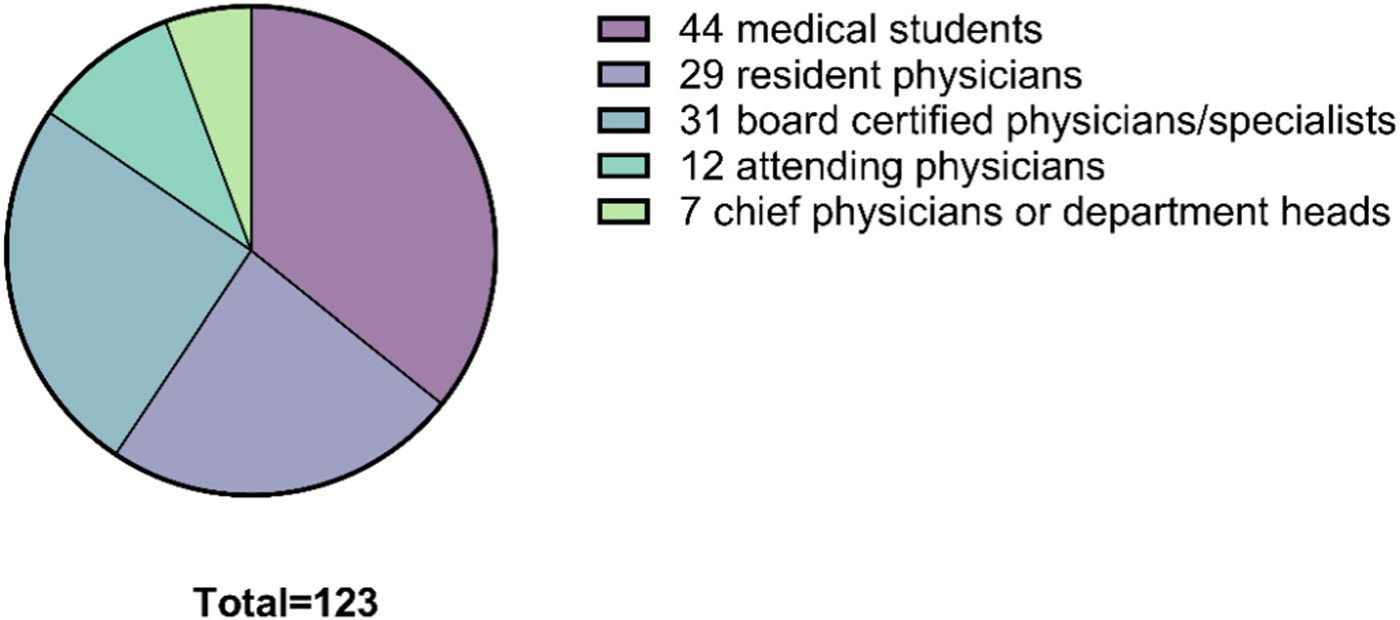
Professional stage of study participants. Pie chart showing the distribution of participants (*N* = 123) by professional stage.

Regarding medical specialization, the majority (*n* = 67, 54.5%) reported internal medicine as their field. Among these, 3 indicated nephrology as their subspecialty. Additionally, 6 participants (4.9%) were general practitioners, 44 (35.8%) had no specialization (i.e., were medical students), and 6 (4.9%) reported other fields or did not specify. An overview of participants’ characteristics is provided in Table 1 and Fig. 2.

**Table 1:** Baseline characteristics of the entire cohort. Overview of participant characteristics, including gender, professional stage, and field of specialization.

***Sex – No.***		
Female	56	(45.5%)
Male	59	(48%)
Not reported	8	(6.5%)
***Medical Education and Professional Stages – No.***		
Medical student	44	(35.8%)
Resident physician	29	(23.6%)
Board certified physician / specialist	31	(25.2%)
Attending physician	12	(9.8%)
Chief physician / Department head	7	(5.7%)
***Field of Specialization – No.***		
Internal Medicine (w/ or w/o subspecializing)	67	(54.5%)
General Medicine	6	(4.9%)
Others or not specified	6	(4.9%)
No specialization yet (i.e., medical student)	44	(35.8%)

### Participants’ performance

Human participants achieved an overall median score of 47% (IQR: 40%–67%) the corresponding mean score was 7.3 ± 2.9 out of 15 possible points. For consistency, all participant scores are reported as percentages of the maximum attainable score (15 points).

Performance varied by professional stage. Specialists attained the highest median score at 53% (IQR: 40%–60%). Residents, attending physicians, and chief physicians had a median score of 47%, with differing interquartile ranges (residents: 40%–70%; attending physicians: 33%–47%; chief physicians: 40%–67%). Medical students recorded the lowest median at 43% (IQR: 22%–67%) and exhibited the widest performance variability.

**Fig. 3:**
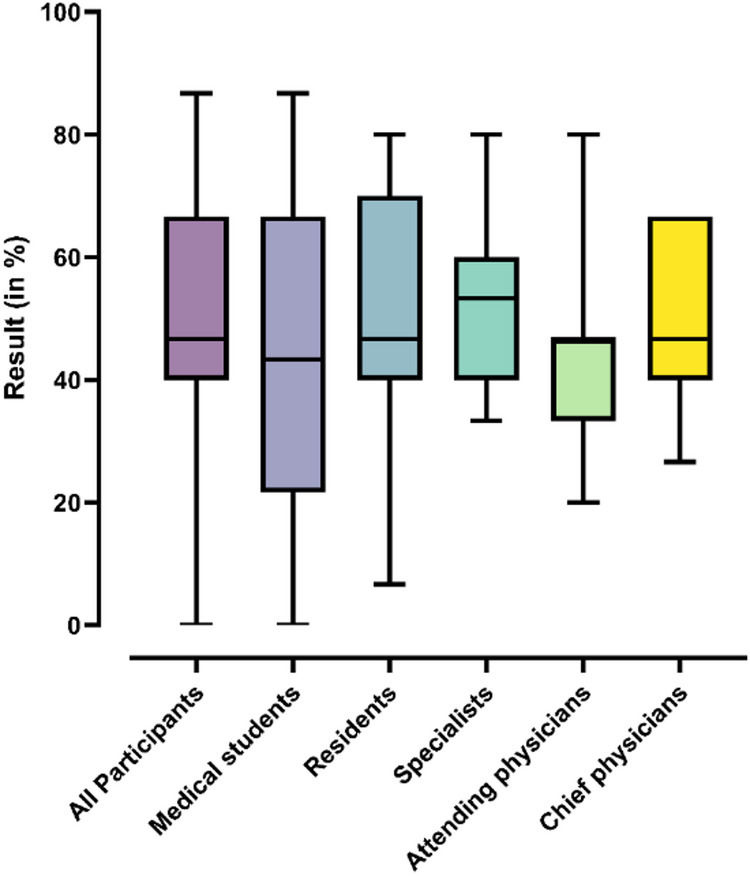
Distribution of participants’ scores by professional stage, shown as percentages of the maximum achievable score. The median score (horizontal line within each box) was highest among specialists (53%) and lowest among medical students (43%). Score variability was greatest among medical students, whereas attending physicians demonstrated the most consistent performance. No statistically significant differences were found between groups (Kruskal–Wallis, *p* = 0.64).

A Kruskal–Wallis test indicated no statistically significant differences between groups (*p* = 0.64; Fig. 3).

### Performance of LLMs and comparison with humans

On average, the LLMs achieved 13.5 (± 1.5) points (90%). The top-performing LLMs (ChatGPT 4o, Gemini 2.0 Flash, DeepSeek V3, and ChatGPT o3-mini-high) each scored 15/15 points (100%), while the lowest-scoring models (ChatGPT 4, ChatGPT 4.5, and ChatGPT 4o-mini) scored 11 points (73%). DeepSeek R1, Mistral Small 3.1, Claude 3.7, ChatGPT o3-mini (reasoning), and Gemini 2.5 Pro Experimental each scored 14 points (93%), while Grok-3 achieved 13 points (87%). In an exploratory extension, each model was tested using three distinct prompts to assess the impact of prompt formulation on performance, with most models demonstrating stable results across conditions. Notably, Mistral Small 3.1 achieved a perfect score (15 out of 15 points) when the prompt was modified by instructing to “respond as if it were an experienced physician in a life-or-death scenario”.

**Fig. 4:**
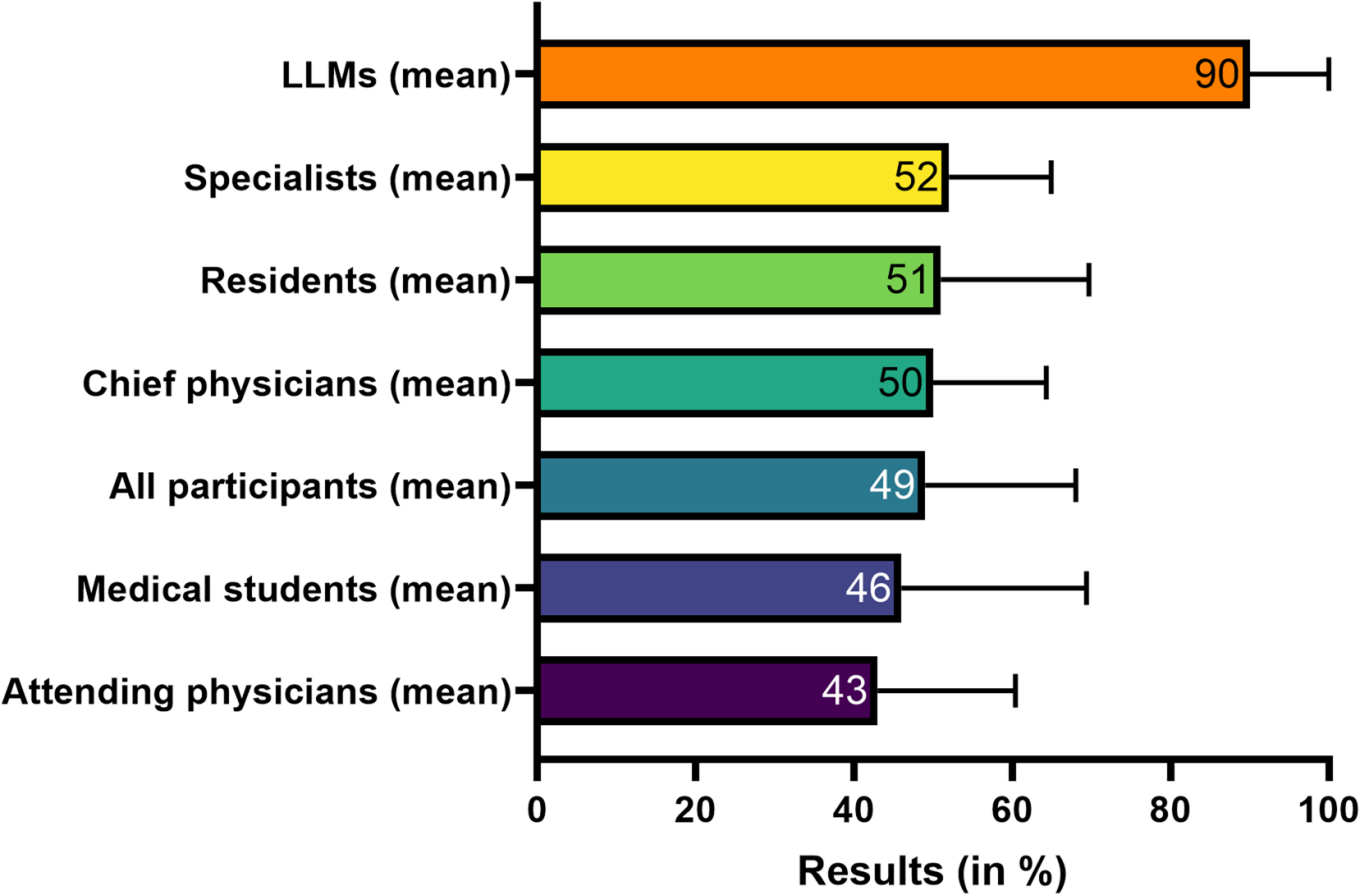
Comparison of overall performance between LLMs (mean across all models) and human participant subgroups. Scores are expressed as percentages of the maximum attainable score (15 points). Bars represent group means, and error bars indicate standard deviations.

LLMs outperformed human participants on average. However, 20 participants (16%) achieved at least 11 points (73%), matching or exceeding the three lowest-scoring LLMs (ChatGPT 4, ChatGPT 4.5, and ChatGPT 4o-mini) used in this study. Among these, 10 participants (8%) scored ≥ 80%, surpassing these models, and one participant achieved 87%, equaling Grok-3. Notably, the collective expectation among participants was that the LLMs would achieve an average score of 13.4 (± 2.5) points (89%). An overview of aggregated group means is presented in Fig. 4, while Supplementary Figure S3 provides a detailed breakdown of performance for each LLM in comparison with human subgroups.

## Response times

Overall, the LLMs completed the task in a fraction of the time required by human participants. For example, ChatGPT completed the test and generated its responses in approximately 0.5 minutes, markedly faster than the human completion time of 7.3 ± 2.5 minutes.

## Discussion

In this cross-sectional study, we demonstrated that a broad spectrum of currently available, publicly accessible LLMs outperformed medical students and physicians in answering structured multiple-choice questions related to AKI. On average, the LLMs exhibited a superior ability to discern and recall factual knowledge while completing the task in significantly less time.

AKI is a frequent and serious condition in emergency care and hospital settings, associated with increased morbidity, mortality, and a heightened risk of developing CKD. Prompt and accurate recognition of AKI, along with appropriate diagnostics and state-of-the-art management, is therefore essential for optimal clinical care^3,33^. The observation that several “participating” LLMs achieved perfect or near-perfect scores on guideline-based AKI case vignettes, whereas no human participant reached a full score, underscores their potential as rapid and accessible tools for standardized knowledge assessment in nephrology and internal medicine. Within the human cohort, we found no statistically significant differences between training stages, ranging from medical students to senior physicians. The absence of variation likely reflects small subgroup sizes, considerable within-group variability, and the informal congress setting rather than true equivalence of knowledge levels.

It should be emphasized, however, that these results reflect factual knowledge recall under standardized test conditions, not real-world decision-making or patient outcomes. They should therefore be regarded as exploratory evidence of performance differences rather than proof of clinical effectiveness.

While LLMs demonstrated strong performance in structured test formats, it is critical not to overlook their inherent limitations. At present, these systems remain susceptible to so-called hallucinations, defined as outputs that are factually incorrect, fabricated, or misleading yet presented in a coherent and persuasive manner^34,35^. If applied unchecked in clinical care, such errors could in extreme cases lead to serious consequences for patient care^36^. Several studies in the medical domain have documented that LLMs may generate inaccurate or misleading clinical statements, underscoring the unpredictability and potential risks of these phenomena^37–39^. This vulnerability may be particularly problematic in complex, ambiguous, or rare clinical scenarios, where guideline-based knowledge may be insufficient. Consequently, AI-generated content must always be critically reviewed and should never be used as a substitute for medical validation. Human oversight remains mandatory.

Although not a central aim of this study, we observed some variation in performance across prompt formulations. In an exploratory observation, the “Mistral Small 3.1” model achieved a higher score when instructed to respond as an experienced physician in a life-or-death scenario. However, because several models from the original study were no longer accessible in their April 2025 versions and others had since been updated by their providers, these exploratory prompt-related observations should be interpreted with caution.

While exploratory, our findings align with emerging literature on prompt engineering in medical AI, suggesting that prompt strategy may affect output quality^40,41^. In clinical applications, this raises important questions about whether models should be pre-tuned for specific domains or dynamically guided depending on context, acuity, and intended use. Recent proof-of-concept studies support this concept, suggesting that retrieval-augmented and disease-specific LLM frameworks may enhance factual accuracy, consistency, and interpretability in clinical settings such as hepatology and sepsis, although safety limitations remain and further refinement is needed before broader clinical implementation^42,43^. This underscores the need for systematic investigations under controlled conditions using version-locked models to ensure reproducibility and comparability, while also acknowledging the inherent tension between such version stability and the continuous evolution of these systems.

Beyond high accuracy, LLMs demonstrated remarkable speed, completing the set of cognitively demanding tasks in seconds, compared to several minutes required by human participants. This rapid response time, combined with broad availability and low marginal costs, positions LLMs as promising tools for rapid knowledge retrieval and assessment. They may offer particular value in time- and resource-constrained settings such as emergency departments, night shifts, or rural clinics. Their speed, availability, and scalability suggest considerable potential to support knowledge access and assist healthcare professionals in managing information more efficiently.

Despite these advantages, it is crucial to emphasize that LLMs lack essential human qualities that are central to clinical practice. They are not present, i.e., physically in the same room with the patient. Furthermore, they cannot observe physical cues, perceive nuance in patient interaction, or express empathy and emotional awareness. These are elements that are foundational to high-quality, person-centered care. Medicine is not solely about factual accuracy; it is also about building trust, understanding suffering, and making context-sensitive decisions. This perspective is supported by evidence showing that empathic, relationship-centered care improves treatment adherence and patient outcomes^44–46^.

Ethical and legal concerns also must be carefully addressed, with ultimate responsibility for clinical decisions remaining with trained human medical professionals. Even when an LLM provides correct information, it is the physician who contextualizes and integrates that knowledge into a broader clinical picture, as current LLMs lack the capability for safe and autonomous clinical decision-making^47^. At present, only the concerted efforts of healthcare professionals, ethicists, and legal experts can help ensure that AI in medicine develops in a reliable, trustworthy, and sustainable manner^48^.

Building on these considerations, the safe integration of LLMs into clinical workflows requires several safeguards. First, their role should remain limited to supportive functions, such as knowledge retrieval and decision assistance, with outputs clearly identifiable as AI-generated, in line with international guidance that emphasizes transparency as a prerequisite for accountability^49–51^. Second, because publicly available LLMs are continuously updated by their providers, their performance may shift over time^52^. Regular benchmarking against clinical guidelines and external validation in diverse settings are therefore essential to detect errors and safeguard reliability^53^. Third, strong ethical and legal frameworks, with particular attention to data protection, are necessary to ensure responsible and sustainable implementation in healthcare^54–56^. Finally, human oversight must remain mandatory, with physicians retaining ultimate responsibility for diagnostic and therapeutic decisions^49,50,57,58^.

### Limitations

Several limitations should be taken into account when interpreting our findings. First, while the study was conducted at one of Europe’s largest congresses of internal medicine using an AKI-focused questionnaire, it did not specifically target nephrology subspecialists. Nonetheless, nephrology forms an integral part of internal medicine, and AKI represents a highly prevalent and clinically significant condition that is addressed across all subspecialties. Accordingly, solid knowledge in this area was reasonably expected from internists regardless of specific subspecialty focus. Second, the test was completed at a self-service station that was not continuously overseen, allowing for potential external influences such as informal collaboration or online searches. Third, participant motivation and concentration may have varied, as answering questions in a busy conference setting differs substantially from high-stakes scenarios, such as formal examinations or real-world clinical encounters with patients in need. Fourth, although the questionnaire was guideline-based and underwent internal expert review within the study team, it was not formally validated. Fifth, the study employed a single cross-sectional design without long-term follow-up or replication in external cohorts;thus, the results should therefore be regarded as exploratory rather than definitive. Sixth, the assessment was limited to 15 multiple-choice questions and two case vignettes. While this format enabled a standardized proof-of-concept comparison, it does not reflect the full breadth of nephrology knowledge or capture higher-order clinical reasoning and patient outcomes. The findings should thus be interpreted strictly as a measure of factual knowledge recall and discernment under specific test conditions. Finally, the study was exploratory in nature and not powered for hypothesis testing; subgroup analyses should therefore be interpreted with caution.

## Conclusion

Current LLMs outperformed medical professionals in structured AKI knowledge assessments, delivering rapid and accurate outputs. While these capabilities underscore their potential as scalable tools for clinical knowledge support, limitations such as residual factual errors, lack of contextual reasoning, and dynamic performance variability demand caution. At present, their role in routine clinical practice remains undefined;crucially, accountable human judgment, clinical expertise, and empathy remain indispensable and irreplaceable components of patient care.

## Supplementary Information

Below is the link to the electronic supplementary material.

Supplementary Material 1

## Data Availability

All relevant data are provided in the article. Additional data can be provided by the corresponding author on reasonable request.

## Abbreviations

AI: artificial intelligence
AKI: acute kidney injury
API: application programming interface
CKD: chronic kidney disease
IQR: inter quartile range
LLM: large language model
ML: machine learning
SD: standard deviation
