# Supplementary material for: Potential of large language models for rapid clinical information support: evidence from acute kidney injury knowledge testing
Source: Sci Rep. 2026 Apr 2;16:11224. doi: 10.1038/s41598-026-46846-7 (PMC13047043; doi:10.1038/s41598-026-46846-7)
Supplement: Supplementary file 1 — Supplementary Material 1 [file 41598_2026_46846_MOESM1_ESM.docx]

**Supplementary Appendix**

**Potential of Large Language Models for Rapid Clinical Information Support: Evidence from Acute Kidney Injury Knowledge Testing**

**Supplementary Table S1.** Overview of included large language models (LLMs) tested (April 2025).

| **Model/version (as provided by developer)** |
| --- |
| ChatGPT 4o |
| ChatGPT 4o-mini |
| ChatGPT 4.5 |
| ChatGPT 4 |
| ChatGPT o3-mini-high |
| ChatGPT o3-mini (reasoning) |
| Claude 3.7 |
| Gemini 2.0 Flash |
| Gemini 2.5 Pro Experimental |
| Mistral Small 3.1 |
| DeepSeek V3-0324 |
| DeepSeek R1 |
| Grok-3 |

**Supplementary File S2.** Questionnaire: LLM vs Human Knowledge Assessment.

## Introductory Questions

Age: _________

Gender: male / female / non-binary

Professional Role / Level of Training:

- Medical student

- Resident physician

- Board-certified specialist

- Attending physician

- Chief physician

- Other: _________

If you are not a medical student, please specify your medical specialty: _______________

## Part I – Case 1

(Questions 1–5 are partially based on the case description below.)

Case 1: A 72-year-old woman presents to the emergency department with loss of appetite, nausea, diarrhea, vomiting, and fever. Symptoms have been present for 3 days. The diarrhea is non-bloody, but several family members have similar symptoms. Past medical history includes breast cancer and arterial hypertension. The patient is admitted for monitoring and treatment. On the following day, her serum creatinine increases from 0.8 mg/dL to 1.4 mg/dL.

**Question 1: According to KDIGO, on which parameters is the classification of acute kidney injury (AKI) based, and how many stages are there?**

- A. 5 stages; serum creatinine, cystatin C, and urine output
- B. 4 stages; serum creatinine and urine creatinine
- C. 4 stages; serum creatinine, urine creatinine, and urine output
- D. 3 stages; serum creatinine, cystatin C, and urine output
- **E. 3 stages; serum creatinine and urine output**

**Question 2: What KDIGO stage of AKI is present in this patient?**

- **A. Stage 1**
- B. Stage 2
- C. Stage 3
- D. Stage 4
- E. Stage 5

**Question 3: What is the most common order of etiologies for AKI?**

- A. Postrenal > prerenal > intrarenal
- B. Postrenal > intrarenal > prerenal
- C. Intrarenal > postrenal > intrarenal
- **D. Prerenal > intrarenal > postrenal**
- E. Prerenal > postrenal > intrarenal

**Question 4: Which statement is NOT correct? An ACE inhibitor…**

- A. Reduces glomerular filtration pressure
- **B. Causes vasodilation of the afferent arteriole**
- C. Was developed based on snake venom research
- D. Reduces proteinuria
- E. Reduces aldosterone release

**Question 5: Which of the following is NOT an indication for acute dialysis?**

- A. Hyperkalemia
- B. Hypervolemia
- **C. Elevated serum creatinine**
- D. Metabolic acidosis
- E. Uremia

## Part II – Case 2

(Questions 6–15 are partially based on the case description below.)

Case 2: A 51-year-old man presents with progressive deterioration in general condition, loss of appetite, pruritus, fatigue, leg edema, and decreased urine output. He reports cola-colored urine and persistent crusting nasal discharge. He has no known prior medical conditions. Laboratory testing reveals serum creatinine of 6.3 mg/dL.

Blood gas results: pH: 7.298; pO₂: 48.9 mmHg; pCO₂: 34.9 mmHg; Base excess: −7.7 mmol/L; HCO₃⁻: 17.9 mmol/L; potassium: 7.2 mmol/L; sodium: 134 mmol/L; lactate: 0.7 mmol/L.

**Question 6: Which acid–base disturbance is present?**

- A. Respiratory acidosis with metabolic compensation
- B. Respiratory acidosis without compensation
- C. Mixed respiratory and metabolic acidosis
- **D. Metabolic acidosis with partial respiratory compensation**
- E. Metabolic acidosis without compensation

**Question 7: The blood gas analysis also shows hyperkalemia (7.2 mmol/L). Which ECG changes are typically associated with hyperkalemia?**

- A. Tachycardia and U wave
- B. Bradycardia and U wave
- C. Tachycardia and shortened QT interval
- **D. Bradycardia and peaked T wave**
- E. Bradycardia and shortened P wave

**Question 8: Which drug does NOT lower serum potassium?**

- **A. Glycopyrronium bromide**
- B. Insulin
- C. Fenoterol
- D. Sodium bicarbonate
- E. Torasemide

**Question 9: Which combination of findings in urinalysis is typical for nephritis?**

- **A. Proteinuria and hematuria**
- B. Proteinuria and ketonuria
- C. Hematuria and glucosuria
- D. Acidic pH and hematuria
- E. Proteinuria and glucosuria

**Question 10: Which urinary sediment findings suggest glomerular injury?**

- A. Hyaline casts and erythrocyte casts
- B. Leukocyte casts and erythrocyte casts
- C. Leukocyte casts and acanthocytes
- D. Granular casts and squamous epithelial cells
- **E. Erythrocyte casts and acanthocytes**

**Question 11: Which symptom is NOT part of the nephrotic syndrome?**

- **A. Hypertension**
- B. Proteinuria
- C. Hypoproteinemia
- D. Hyperlipoproteinemia
- E. Peripheral edema

**Question 12: Which triad is typical for nephritic syndrome?**

- A. Leukocyturia, macroscopic hematuria, and edema
- B. Macroscopic hematuria, edema, and proteinuria
- C. Leukocyturia, edema, and hypertension
- **D. Microscopic hematuria, edema, and hypertension**
- E. Proteinuria, leukocyturia, and edema

**Question 13: Which histologic finding is LEAST typical for active glomerulonephritis?**

- A. Extracapillary proliferation (crescents)
- **B. Interstitial edema formation**
- C. Infiltration by neutrophils and macrophages
- D. Necrosis
- E. Mesangial cell proliferation

**Question 14: The presence of which antibody is NOT typical for rapidly progressive glomerulonephritis?**

- A. c-ANCA
- B. p-ANCA
- C. Anti-GBM antibody
- D. ANA
- **E. Ro/SS-A antibody**

**Question 15: What does the abbreviation ANCA stand for?**

- **A. Anti-neutrophil cytoplasmic antibodies**
- B. Anti-neutrophil circulating antibodies
- C. Anti-nuclear cytoplasmic antibodies
- D. Anti-nuclear circulating antibodies
- E. Anti-nephrin circulating antibodies

**Supplementary Figure S3 | Comparison of overall performance between LLMs) and human participants.** Blue bars represent individual LLM scores; orange bars show the mean scores of participant subgroups by professional stage. All scores are expressed as percentages of the maximum achievable score (15 points).
